# (*E*)-*N*-(6-Chloro-3-pyridylmeth­yl)-*N*-ethyl-*N*′-methyl-2-nitro­ethyl­ene-1,1-diamine

**DOI:** 10.1107/S1600536808013317

**Published:** 2008-05-14

**Authors:** Liang-Zhong Xu, Zhi Yang, Xu Yi, Guang-Wei An

**Affiliations:** a College of Chemistry and Molecular Engineering, Qingdao University of Science and Technology, Qingdao 266042, People’s Republic of China

## Abstract

In the title compound, C_11_H_15_ClN_4_O_2_, the amino group is involved in intra- and inter­molecular N—H⋯O hydrogen bonds. The former contributes to the mol­ecular conformation, while the latter link the mol­ecules into centrosymmetric dimers. The crystal structure also exhibits weak inter­molecular C—H⋯O inter­actions.

## Related literature

For the properties of neonicotinoid insecticides, see: Wang *et al.* (2001 ▶); Isao *et al.* (1993 ▶). For related crystal structures, see: Jiang *et al.* (2007 ▶); Xia *et al.* (2007 ▶).
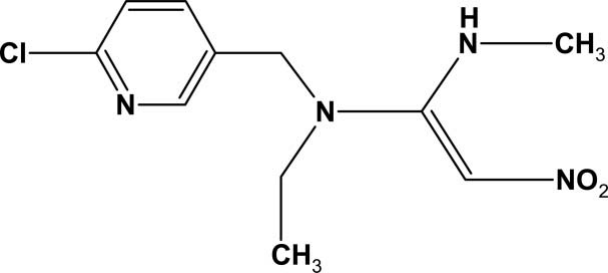


## Experimental

### 

#### Crystal data

C_11_H_15_ClN_4_O_2_
*M* *_r_* = 270.72
Monoclinic, 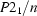

*a* = 7.7252 (15) Å
*b* = 7.9281 (16) Å
*c* = 20.787 (4) Å
β = 92.34 (3)°
*V* = 1272.0 (4) Å^3^
*Z* = 4
Mo *K*α radiation
μ = 0.30 mm^−1^
*T* = 113 (2) K
0.14 × 0.12 × 0.04 mm

#### Data collection

Rigaku Saturn diffractometer
Absorption correction: multi-scan (*CrystalClear*; Rigaku, 2005 ▶) *T*_min_ = 0.959, *T*_max_ = 0.988
7120 measured reflections
2238 independent reflections
1970 reflections with *I* > 2σ(*I*)
*R*_int_ = 0.031

#### Refinement

*R*[*F* ^2^ > 2σ(*F* ^2^)] = 0.033
*wR*(*F* ^2^) = 0.089
*S* = 1.05
2238 reflections
165 parameters
H-atom parameters constrained
Δρ_max_ = 0.33 e Å^−3^
Δρ_min_ = −0.34 e Å^−3^

### 

Data collection: *CrystalClear* (Rigaku, 2005 ▶); cell refinement: *CrystalClear*; data reduction: *CrystalClear*; program(s) used to solve structure: *SHELXTL* (Sheldrick, 2008 ▶); program(s) used to refine structure: *SHELXTL*; molecular graphics: *SHELXTL*; software used to prepare material for publication: *SHELXTL*.

## Supplementary Material

Crystal structure: contains datablocks global, I. DOI: 10.1107/S1600536808013317/cv2407sup1.cif

Structure factors: contains datablocks I. DOI: 10.1107/S1600536808013317/cv2407Isup2.hkl

Additional supplementary materials:  crystallographic information; 3D view; checkCIF report

## Figures and Tables

**Table 1:** Hydrogen-bond geometry (Å, °)

*D*—H⋯*A*	*D*—H	H⋯*A*	*D*⋯*A*	*D*—H⋯*A*
N3—H3*A*⋯O1	0.86	2.12	2.6376 (16)	118
N3—H3*A*⋯O1^i^	0.86	2.38	3.0778 (17)	138
C6—H6*A*⋯O1^ii^	0.97	2.58	3.508 (2)	160
C3—H3⋯O2^iii^	0.93	2.50	3.1101 (19)	123

Symmetry codes: (i) 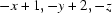; (ii) 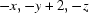; (iii) 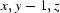.

**Fig. 1.**
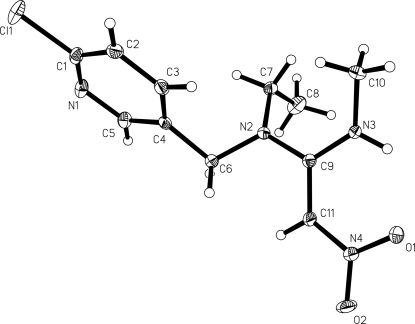
The molecular structure of the title compound with the atom-labeling scheme. Displacement ellipsoids are drawn at the 40% probability level.

## supplementary crystallographic information

### Comment

Nitenpyram is a new chloro-nicotine type insecticide (Wang *et al.*, 2001) possessing a wide spectum of useful properties (Isao *et al.*, 1993). We report here the crystal structure of the title compound (I).

In (I) (Fig. 1), all bond lengths and angles are in agreement with those reported for the related structures (Jiang *et al.*, 2007; Xia *et al.*, 2007). The pyridine ring and plane N2/N3/C9/C11 form a dihedral angle of 48.8 (2)°. The amino group is involved in intra- and intermolecular N-H···O hydrogen bonds (Table 1). The intermolecular N-H···O hydrogen bonds  link the molecules into centrosymmetric dimers. The crystal packing exhibits also weak intermolecular C—H···O interactions (Table 1).

### Experimental

A solution comprising *N*-((6-chloropyridin-3-yl)methyl)ethanamine(0.1 mol) in trichloromethane(30 ml) was slowly added from a dropping-funnel to a mixture of 1,1,1-trichloro-2-nitro-ethane(0.15 mol), trichloromethane(30 ml) and an aqueous solution of sodium carbonate(40%, 53 g) in a flask equipped with stirrer and reflux condenser. After the mixture was stirred for 1 h while maintaining the temperature at 273–280k, an aqueous solution of methylamine(30%, 30 g) was then added dropwise, followed by a two hours stirring at room temperature. The reaction mixture were extracted with trichloromethane, the title compound (16.2 g, yield 60%) could be afforded after ethyl oxide was added to the concentration remnants on cooling. The single-crystal suitable for X-ray measurements was obtained by recrystallization from trichloromethane-ethyl acetate(1:1) at room temperature.

### Refinement

H atoms were positioned geometrically and allowed to ride on their parent atoms, with N—H and C—H distances of 0.86 and 0.93–0.97 Å, respectively, and with *U*_iso_(H) = 1.2*U*_eq_(C, N) for the aryl, methylene and N H atoms and 1.5*U*_eq_(C) for the methyl H atoms.

### Crystal data

**Table:**

C_11_H_15_ClN_4_O_2_	*F*_000_ = 568
*M**_r_* = 270.72	*D*_x_ = 1.414 Mg m^−^^3^
Monoclinic, *P*2_1_/*n*	Mo *K*α radiation λ = 0.71073 Å
Hall symbol: -P 2yn	Cell parameters from 3653 reflections
*a* = 7.7252 (15) Å	θ = 2.0–27.9º
*b* = 7.9281 (16) Å	µ = 0.30 mm^−^^1^
*c* = 20.787 (4) Å	*T* = 113 (2) K
β = 92.34 (3)º	Block, yellow
*V* = 1272.0 (4) Å^3^	0.14 × 0.12 × 0.04 mm
*Z* = 4	

### Data collection

**Table:**

Rigaku Saturn diffractometer	2238 independent reflections
Radiation source: rotating anode	1970 reflections with *I* > 2σ(*I*)
Monochromator: confocal	*R*_int_ = 0.031
*T* = 113(2) K	θ_max_ = 25.0º
ω scans	θ_min_ = 2.8º
Absorption correction: multi-scan(CrystalClear; Rigaku, 2005)	*h* = −9→5
*T*_min_ = 0.959, *T*_max_ = 0.988	*k* = −8→9
7120 measured reflections	*l* = −24→23

### Refinement

**Table:**

Refinement on *F*^2^	Secondary atom site location: difference Fourier map
Least-squares matrix: full	Hydrogen site location: inferred from neighbouring sites
*R*[*F*^2^ > 2σ(*F*^2^)] = 0.033	H-atom parameters constrained
*wR*(*F*^2^) = 0.089	*w* = 1/[σ^2^(*F*_o_^2^) + (0.053*P*)^2^ + 0.2942*P*] where *P* = (*F*_o_^2^ + 2*F*_c_^2^)/3
*S* = 1.05	(Δ/σ)_max_ < 0.001
2238 reflections	Δρ_max_ = 0.33 e Å^−^^3^
165 parameters	Δρ_min_ = −0.34 e Å^−^^3^
Primary atom site location: structure-invariant direct methods	Extinction correction: none

### Special details

**Table:**

Geometry. All e.s.d.'s (except the e.s.d. in the dihedral angle between two l.s. planes) are estimated using the full covariance matrix. The cell e.s.d.'s are taken into account individually in the estimation of e.s.d.'s in distances, angles and torsion angles; correlations between e.s.d.'s in cell parameters are only used when they are defined by crystal symmetry. An approximate (isotropic) treatment of cell e.s.d.'s is used for estimating e.s.d.'s involving l.s. planes.
Refinement. Refinement of *F*^2^ against ALL reflections. The weighted *R*-factor *wR* and goodness of fit *S* are based on *F*^2^, conventional *R*-factors *R* are based on *F*, with *F* set to zero for negative *F*^2^. The threshold expression of *F*^2^ > σ(*F*^2^) is used only for calculating *R*-factors(gt) *etc*. and is not relevant to the choice of reflections for refinement. *R*-factors based on *F*^2^ are statistically about twice as large as those based on *F*, and *R*- factors based on ALL data will be even larger.

### Fractional atomic coordinates and isotropic or equivalent isotropic displacement parameters (Å^2^)

**Table:**

	*x*	*y*	*z*	*U*_iso_*/*U*_eq_	
Cl1	−0.46834 (6)	0.13093 (5)	0.15973 (2)	0.03277 (16)	
O1	0.31814 (14)	1.08651 (13)	−0.00180 (6)	0.0256 (3)	
O2	0.11880 (15)	1.26510 (13)	0.02461 (6)	0.0297 (3)	
N1	−0.37165 (16)	0.43140 (16)	0.19759 (6)	0.0218 (3)	
N2	0.10858 (15)	0.74385 (14)	0.12607 (6)	0.0154 (3)	
N3	0.34655 (15)	0.80473 (14)	0.06527 (6)	0.0164 (3)	
H3A	0.4171	0.8837	0.0555	0.020*	
N4	0.19059 (16)	1.12460 (14)	0.03211 (6)	0.0187 (3)	
C1	−0.34845 (19)	0.31625 (19)	0.15333 (7)	0.0194 (3)	
C2	−0.23838 (19)	0.33022 (19)	0.10271 (8)	0.0193 (3)	
H2	−0.2261	0.2429	0.0734	0.023*	
C3	−0.14779 (18)	0.47891 (18)	0.09762 (7)	0.0183 (3)	
H3	−0.0727	0.4941	0.0642	0.022*	
C4	−0.16921 (18)	0.60648 (17)	0.14276 (7)	0.0157 (3)	
C5	−0.28034 (19)	0.57502 (19)	0.19164 (7)	0.0193 (3)	
H5	−0.2931	0.6584	0.2225	0.023*	
C6	−0.07572 (18)	0.77266 (17)	0.13772 (7)	0.0170 (3)	
H6A	−0.1280	0.8384	0.1027	0.020*	
H6B	−0.0863	0.8360	0.1773	0.020*	
C7	0.20362 (19)	0.64947 (18)	0.17777 (7)	0.0177 (3)	
H7A	0.2889	0.5772	0.1586	0.021*	
H7B	0.1228	0.5777	0.1996	0.021*	
C8	0.2939 (2)	0.7633 (2)	0.22653 (9)	0.0301 (4)	
H8A	0.3829	0.8259	0.2062	0.045*	
H8B	0.3449	0.6965	0.2608	0.045*	
H8C	0.2115	0.8402	0.2436	0.045*	
C9	0.19636 (18)	0.85310 (17)	0.08850 (7)	0.0142 (3)	
C10	0.4040 (2)	0.63299 (18)	0.05466 (8)	0.0206 (3)	
H10A	0.4731	0.5955	0.0913	0.031*	
H10B	0.4718	0.6292	0.0170	0.031*	
H10C	0.3050	0.5607	0.0486	0.031*	
C11	0.12900 (18)	1.01529 (17)	0.07620 (7)	0.0169 (3)	
H11	0.0359	1.0503	0.0999	0.020*	

### Atomic displacement parameters (Å^2^)

**Table:**

	*U*^11^	*U*^22^	*U*^33^	*U*^12^	*U*^13^	*U*^23^
Cl1	0.0374 (3)	0.0288 (2)	0.0319 (3)	−0.01993 (18)	0.00009 (19)	0.00218 (17)
O1	0.0213 (6)	0.0257 (6)	0.0307 (7)	0.0037 (5)	0.0121 (5)	0.0098 (5)
O2	0.0367 (7)	0.0148 (5)	0.0384 (8)	0.0088 (5)	0.0118 (6)	0.0096 (5)
N1	0.0195 (6)	0.0264 (7)	0.0195 (7)	−0.0078 (6)	0.0008 (5)	0.0030 (6)
N2	0.0126 (6)	0.0143 (6)	0.0191 (7)	0.0006 (5)	0.0006 (5)	0.0041 (5)
N3	0.0137 (6)	0.0119 (6)	0.0241 (7)	−0.0011 (5)	0.0041 (5)	0.0007 (5)
N4	0.0191 (7)	0.0145 (6)	0.0226 (7)	0.0014 (5)	0.0033 (5)	0.0023 (5)
C1	0.0171 (7)	0.0202 (7)	0.0203 (8)	−0.0049 (6)	−0.0052 (6)	0.0054 (6)
C2	0.0197 (7)	0.0172 (7)	0.0208 (8)	0.0025 (6)	−0.0012 (6)	0.0005 (6)
C3	0.0149 (7)	0.0202 (8)	0.0199 (8)	0.0017 (6)	0.0028 (6)	0.0032 (6)
C4	0.0115 (7)	0.0170 (7)	0.0183 (8)	0.0018 (6)	−0.0023 (6)	0.0040 (6)
C5	0.0179 (7)	0.0218 (7)	0.0181 (8)	−0.0025 (6)	0.0003 (6)	−0.0006 (6)
C6	0.0147 (7)	0.0154 (7)	0.0211 (8)	0.0012 (6)	0.0033 (6)	0.0022 (6)
C7	0.0171 (7)	0.0176 (7)	0.0183 (8)	0.0002 (6)	−0.0006 (6)	0.0053 (6)
C8	0.0348 (9)	0.0279 (9)	0.0267 (10)	−0.0077 (7)	−0.0094 (7)	0.0053 (7)
C9	0.0141 (7)	0.0134 (7)	0.0148 (8)	−0.0016 (6)	−0.0018 (6)	−0.0010 (5)
C10	0.0200 (8)	0.0165 (7)	0.0252 (9)	0.0052 (6)	0.0026 (7)	−0.0012 (6)
C11	0.0163 (7)	0.0150 (7)	0.0197 (8)	0.0005 (6)	0.0056 (6)	0.0008 (6)

### Geometric parameters (Å, °)

**Table:**

Cl1—C1	1.7447 (15)	C4—C5	1.379 (2)
O1—N4	1.2711 (16)	C4—C6	1.508 (2)
O2—N4	1.2512 (16)	C5—H5	0.9300
N1—C1	1.314 (2)	C6—H6A	0.9700
N1—C5	1.3478 (19)	C6—H6B	0.9700
N2—C9	1.3650 (18)	C7—C8	1.507 (2)
N2—C6	1.4717 (17)	C7—H7A	0.9700
N2—C7	1.4796 (19)	C7—H7B	0.9700
N3—C9	1.3313 (18)	C8—H8A	0.9600
N3—C10	1.4517 (18)	C8—H8B	0.9600
N3—H3A	0.8600	C8—H8C	0.9600
N4—C11	1.3613 (18)	C9—C11	1.4066 (19)
C1—C2	1.384 (2)	C10—H10A	0.9600
C2—C3	1.377 (2)	C10—H10B	0.9600
C2—H2	0.9300	C10—H10C	0.9600
C3—C4	1.394 (2)	C11—H11	0.9300
C3—H3	0.9300		
			
C1—N1—C5	115.85 (13)	N2—C6—H6B	109.6
C9—N2—C6	120.14 (12)	C4—C6—H6B	109.6
C9—N2—C7	119.54 (12)	H6A—C6—H6B	108.1
C6—N2—C7	114.44 (11)	N2—C7—C8	112.82 (12)
C9—N3—C10	126.98 (12)	N2—C7—H7A	109.0
C9—N3—H3A	116.5	C8—C7—H7A	109.0
C10—N3—H3A	116.5	N2—C7—H7B	109.0
O2—N4—O1	119.49 (12)	C8—C7—H7B	109.0
O2—N4—C11	119.03 (12)	H7A—C7—H7B	107.8
O1—N4—C11	121.48 (12)	C7—C8—H8A	109.5
N1—C1—C2	125.63 (13)	C7—C8—H8B	109.5
N1—C1—Cl1	116.35 (11)	H8A—C8—H8B	109.5
C2—C1—Cl1	118.03 (12)	C7—C8—H8C	109.5
C3—C2—C1	117.18 (14)	H8A—C8—H8C	109.5
C3—C2—H2	121.4	H8B—C8—H8C	109.5
C1—C2—H2	121.4	N3—C9—N2	119.04 (12)
C2—C3—C4	119.65 (14)	N3—C9—C11	121.26 (13)
C2—C3—H3	120.2	N2—C9—C11	119.69 (12)
C4—C3—H3	120.2	N3—C10—H10A	109.5
C5—C4—C3	117.33 (13)	N3—C10—H10B	109.5
C5—C4—C6	121.56 (13)	H10A—C10—H10B	109.5
C3—C4—C6	121.10 (13)	N3—C10—H10C	109.5
N1—C5—C4	124.34 (14)	H10A—C10—H10C	109.5
N1—C5—H5	117.8	H10B—C10—H10C	109.5
C4—C5—H5	117.8	N4—C11—C9	124.57 (13)
N2—C6—C4	110.19 (11)	N4—C11—H11	117.7
N2—C6—H6A	109.6	C9—C11—H11	117.7
C4—C6—H6A	109.6		
			
C5—N1—C1—C2	0.8 (2)	C3—C4—C6—N2	−47.41 (19)
C5—N1—C1—Cl1	−179.06 (11)	C9—N2—C7—C8	58.95 (17)
N1—C1—C2—C3	−1.3 (2)	C6—N2—C7—C8	−94.11 (15)
Cl1—C1—C2—C3	178.57 (11)	C10—N3—C9—N2	23.9 (2)
C1—C2—C3—C4	0.3 (2)	C10—N3—C9—C11	−157.47 (15)
C2—C3—C4—C5	1.0 (2)	C6—N2—C9—N3	−163.24 (13)
C2—C3—C4—C6	−178.39 (13)	C7—N2—C9—N3	45.24 (19)
C1—N1—C5—C4	0.7 (2)	C6—N2—C9—C11	18.2 (2)
C3—C4—C5—N1	−1.6 (2)	C7—N2—C9—C11	−133.36 (14)
C6—C4—C5—N1	177.82 (13)	O2—N4—C11—C9	179.01 (14)
C9—N2—C6—C4	145.80 (13)	O1—N4—C11—C9	−0.4 (2)
C7—N2—C6—C4	−61.31 (16)	N3—C9—C11—N4	11.8 (2)
C5—C4—C6—N2	133.23 (14)	N2—C9—C11—N4	−169.61 (14)

### Hydrogen-bond geometry (Å, °)

**Table:**

*D*—H···*A*	*D*—H	H···*A*	*D*···*A*	*D*—H···*A*
N3—H3A···O1	0.86	2.12	2.6376 (16)	118
N3—H3A···O1^i^	0.86	2.38	3.0778 (17)	138
C6—H6A···O1^ii^	0.97	2.58	3.508 (2)	160
C3—H3···O2^iii^	0.93	2.50	3.1101 (19)	123

Symmetry codes: (i) −*x*+1, −*y*+2, −*z*; (ii) −*x*, −*y*+2, −*z*; (iii) *x*, *y*−1, *z*.
